# Energy Consumption and Cold Hardiness of Diapausing Fall Webworm Pupae

**DOI:** 10.3390/insects13090853

**Published:** 2022-09-19

**Authors:** Lvquan Zhao, Xinmei Wang, Zheng Liu, Alex S. Torson

**Affiliations:** 1 Co-Innovation Center for Sustainable Forestry in Southern China, College of Forestry, Nanjing Forestry University, Nanjing 210037, China; wangxinmei@njfu.edu.cn (X.W.); liuzheng@njfu.edu.cn (Z.L.); 2 USDA-ARS Edward T. Schafer Agricultural Research Center, Biosciences Research Laboratory, Fargo, ND 58102, USA; alex.torson@usda.gov

**Keywords:** *Hyphantria cunea*, diapause, energy consumption, supercooling point, post-diapause fitness

## Abstract

**Simple Summary:**

The fall webworm, *Hyphantria cunea*, a major invasive pest in China, overwinters as diapausing pupae, but seasonal changes in energy consumption and cold hardiness of diapausing pupae are still unclear. In this study, we investigated the seasonal variation in lipid, glycogen, and trehalose content accompanying changes in the supercooling points of diapausing pupae. We found that the energy consumption of *H. cunea* diapausing pupae was dominated by lipid and carbohydrates early in diapause and shifted to glycogen or other energy stores as diapause progressed. We also found that an increase in pupal diapause development time had a significant negative effect on the survival rate of pupae during diapause and the post diapause adult fitness. This information is essential for the development of a theoretical foundation to better understand the overwintering strategy of *H. cunea* and for improving forecasts of the population dynamics according to the thermal conditions in various years.

**Abstract:**

Diapause and cold hardiness are essential components of winter survival for most insects in temperate zones. The fall webworm, *Hyphantria cunea*, overwinters in a pupal diapause. In this study, we investigated the energy consumption and cold hardiness of diapausing pupae. We found that lipid content decreased from October to November and stabilized from November to March. Glycogen content decreased by 61.3% and 52.2% for females and males, respectively, from October to November, and decreased slowly from November to March. We also observed a significant increase in trehalose concentrations as ambient temperatures decreased from October to November and a decrease in trehalose as temperatures increased again in March. We did not observe substantial changes in pupal supercooling points among the dates sampled. In addition, prolonged pupal development time reduced their survival rate and had no significant effect on post-diapause adult body mass and fecundity but reduced egg diameter in females. These results suggest that the energy consumption of *H. cunea* pupae during early diapause depends on lipid and glycogen, while it shifts to depend on glycogen or other energy stores in the mid- and late diapause stages. Our results also suggest that the prolonged development time of diapausing pupae had a negative effect on post-diapause fitness.

## 1. Introduction

Insects are poikilothermic animals, so their metabolic rate fluctuates with changes in ambient temperature and is affected by extreme temperatures [1,2]. To cope with harsh environmental conditions, many insects enter diapause, a seasonal, programmed state of developmental arrest that is induced by environmental factors such as photoperiod, promoting the accrual of energy reserves prior to the onset of the diapause phenotype [3,4,5,6].

Most diapausing insects do not feed, which thus poses a significant energy consumption challenge [7]. Diapause-destined insects prepare for winter by accumulating energy stores, primarily in the form of lipids, during diapause preparation and decreasing metabolic rate during diapause [8]. Although lipids are the primary fuel source during diapause, insects can shift from lipids to other energy reserves at specific stages [7,9]. For example, the flesh fly *Sarcophaga crassipalpis* Macquart, shifts its energy consumption from lipids during early diapause to carbohydrates and other energy substrates later in diapause [10]. Carbohydrates can be used as energy reserves and function as cryoprotectants, responding rapidly to temperature changes to improve insect survival [6,11]. For example, glycogen and trehalose can be rapidly converted to glucose to meet energy requirements and provide a source material for the synthesis of polyhydric alcohols for cold hardiness [7]. Some insect species such as the Eastern spruce budworm *Choristoneura fumiferana* (Clemens) rely heavily on carbohydrates for their energy source throughout diapause [12].

The energy reserves of diapausing insects are not only used to maintain basic body functions, but also provide a source of energy for post-diapause development [7]. Therefore, the degree of energy consumption during diapause can have a significant effect on post-diapause fitness. For example, goldenrod gall flies, *Eurosta solidaginis* Fitch, exposed to warmer temperatures during diapause, have increased energy consumption, resulting in a lower overwintering survival rate and post-diapause adult fecundity [13]. Additionally, a prolonged diapause for the blue orchard bee, *Osmia lignaria* Say, leads to increased lipid consumption and weight loss, ultimately increasing larval mortality and negatively impacting the likelihood of nest establishment [14,15,16,17]. Although energy consumption during diapause in insects is critical, the correlation or trade-off between diapause termination time and post-diapause energetics remains unclear [18].

In addition to energy consumption challenges, an insect in diapause will face the stress of winter, where low temperatures and freezing can cause physiological damage and death. Thus, diapausing insects often become more cold-tolerant [19,20]. Under unfavorable environmental conditions, insects synthesize and accumulate distinct types of low-molecular-weight compounds such as polyols, sugars, and some amino acids [21], which function as cryoprotectants and improve their cold hardiness [22,23]. For example, during diapause development, the pupae of *Phyllonorycter ringoniella* Matsumura gradually accumulate more trehalose, which is used as a cryoprotectant to improve their cold hardiness [24].

The fall webworm, *Hyphantria cunea* (Drury) (Lepidoptera: Arctiidae), a major invasive pest in China, is native to North America and was introduced into Japan around 1970 and was subsequently introduced into China [25]. *Hyphantria cunea* overwinters as diapausing pupae. Li et al., found that under field conditions, overwintering pupae of *H. cunea* in Japan do not develop a high level of cold hardiness; the lipid content of diapause pupae did not change during diapause, while the glycogen content was relatively high from November to December, dropped to low levels from January to March, and then partially recovered in April [26]. The results of Xu et al., showed that the fat content of diapausing pupae from Northwest China decreased gradually during winter and the supercooling point and trehalose showed distinct seasonal variation patterns [27]. Since its introduction to Dandong city (about 40 °N) in 1979, *H. cunea* has now spread to Nanjing city (about 32 °N), Jiangsu province [28], and its expansion is trending southward. Due to geographic and latitudinal gradients in temperature, *H. cunea* will encounter drastically different environmental conditions as it expands its range southward in China. Winter temperatures are the key factor in modulating insect diapause and their cold hardiness characteristic. Therefore, it is necessary to investigate the physiology of diapause and the cold hardiness of *H. cunea* population in the southeast of China.

To successfully overwinter, the pre-diapause *H. cunea* store more lipid and carbohydrates than non-diapausing pupae [29]. However, the change pattern of energy consumption and cold hardiness of diapausing pupae with seasonal change and the effect of diapausing pupae development time on their survival rate and post-diapause fitness are not clear. In this study, we investigated the seasonal variation pattern of lipid and glycogen content, supercooling point and trehalose accompanying the change in the supercooling point of diapausing pupae. Furthermore, we investigated the effects of variation in the developmental time of diapausing pupae on their survival rate and post-diapause fitness. The result of the present study is essential to the development of a theoretical foundation for a better understanding of the overwintering strategy of *H. cunea* pupae.

## 2. Materials and Methods

### 2.1. Insect Collection and Rearing

Final-instar larvae of *H. cunea* were collected in early October 2020 from poplar groves in Chuzhou District, Huai’an City of Jiangsu province and brought back to the laboratory and placed in the greenhouse, where they were placed in plastic containers (30 cm × 20 cm × 15 cm) with poplar leaves (*Populus deltoids*). The front and sides of the plastic containers were perforated to ensure ventilation, while the density of larvae in each container (˂20 individuals/container) was controlled to ensure normal development and survival rate. The leaves were changed once a day and the larvae were checked for pupation when the leaves were changed. *Hyphantria cunea* progress through three generations per year under natural environmental conditions in Huai’an area. Thus, we collected final instar larvae to ensure that larvae would enter diapause after pupation.

### 2.2. Experimental Design

We sexed the pupae according to the presence (female) or absence (male) of a line intersecting the first abdominal sternite on the second day after pupation [30,31], separated them into separate plastic containers (5 cm diam. ×4 cm high), and placed them in a carton to ensure dry conditions. We placed each carton on the veranda outside of the laboratory with a small amount of desiccated cotton over the plastic container and a black plastic bag covering the outside of the carton for shade and protection from rain.

### 2.3. Ambient Temperature

A temperature logger (GM1365, Jumaoyuan Technology Co., Ltd., Shenzhen, China) was placed on the outside veranda of the laboratory where the diapausing pupae were located and recorded the ambient temperature every five minutes.

### 2.4. Supercooling Point

*Hyphantria cunea* overwinter as diapausing pupae beginning at the end of September or early October and emerge as adults at the end of April or early May of the following year. We measured the supercooling point of diapausing pupae on the 28th day of each month from October 2020 to March 2021. The supercooling points were determined by the supercooling determinator (SUN-II intelligent insect-SCP determinator, SUN Company, Jinan, China). The pupae were placed on the probe of the supercooling point determinator. The samples with the probes attached were placed in a refrigerated test chamber, and the temperature of the refrigerated test chamber was reduced from 15 °C to −25 °C at a rate of 0.5 °C per minute. Insect body temperature was recorded every 1.6 s by the instrument.

### 2.5. Biochemical Analysis

#### 2.5.1. Trehalose and Glycogen

We homogenized pupae in a 1.5 mL microcentrifuge tube with 50 µL of 10 % trichloroacetic acid solution and a small amount of quartz sand. The pestle used to homogenize the sample was rinsed with 950 µL of 10% trichloroacetic acid solution. After sufficient shaking, the mixture was centrifuged at 4 °C at 5000 rpm for 5 min. We transferred the supernatant to a 2 mL microcentrifuge tube and dissolved the precipitate in 1000 µL of 10% trichloroacetic acid solution. After centrifuging the dissolved precipitate, we combined this supernatant with the supernatant collected after the first centrifugation and used the residual precipitate to measure glycogen content. We removed 500 µL of sample from the 2 mL tubes and transferred to a 1.5 mL tube containing 1 mL of ethanol and placed in a refrigerator at 4 °C for 16 h. The tube was removed from the refrigerator and the supernatant was centrifuged at 4 °C for 20 min at l0,000 rpm. The upper supernatant was transferred into a 10 mL stoppered graduated test tube and 1 mL of 0.15 mol/L sulfuric acid solution was added, shaken and mixed. The mixture was heated in a boiling water bath for 10 min, cooled under running water and 1 mL of 30% KOH solution was added. After shaking and mixing, the mixture was heated again in a boiling water bath for 10 min and then cooled under running water and used for the determination of trehalose content.

We quantified the glycogen and trehalose content using the anthrone method [32]. The lower precipitate obtained in the above step was fully dissolved in 1 mL of distilled water before determining the content of trehalose. Samples (50 µL of trehalose and glycogen solutions) were mixed with 950 µL of distilled water, and 4 mL of 0.1% anthrone solution (1 g anthrone in 1000 mL 80% sulfuric acid) was added in an ice-water bath. We then heated the samples to 100 °C for 10 min and then cooled under running water. Absorbance was determined at 620 nm, and concentrations were determined by comparison to standards of known glucose concentrations.

#### 2.5.2. Lipid

The lipids of diapausing pupae were extracted as described by Lorenz [33], and the lipid content was determined by the sulphophosphovanillin method. Samples (50 µL) were added to 950 µL sulfuric acid, and then the mixtures were incubated at 100 °C for 10 min. After cooling, 5 mL of 0.2% phosphovanillin in 57% ortho-phosphoric acid was added to the mixtures. Samples were measured against cholesterol standards at 530 nm.

### 2.6. Development Time of Diapausing Pupae and Post-Diapause Adult Fitness

The *H. cunea* diapausing pupae emerged as adults in late April and non-diapausing pupae completed emergence within 20 d after pupation. From 5 February, the diapausing pupae were moved from the outdoor to the dark incubator at 25 ± 1 °C at intervals of 20 d. The adult emergence time and number of adults were observed and recorded daily. After emergence, the adults were kept in an incubator at 25 ± 1 °C with photoperiod 16:8 L:D and their body mass was measured (AL104 Mettler-Toledo; Mettler-Toledo, Zurich, Switzerland, d = 0.0001 g). On the day of female death, the ovaries were dissected under StrREO Discovery V8 Zeiss stereomicroscope and the number of eggs was counted in a buffer-filled wax disc and the egg diameter was measured using the Zen imaging software (version Zen 2, Carl Zeiss, Jena, Germany).

### 2.7. Data Analysis

The experimental data were analyzed in SPSS 22.0 (IBM Inc., New York, NY, USA). The supercooling point, time from pupation to adult emergence and biochemical composition of diapausing pupae, and the body mass, fecundity and egg diameter of post-diapause adult were analyzed using analysis of variance (ANOVA) with post hoc Tukey’s multiple range test. The Kruskal–Wallis test was used to analyze the survival rate of diapausing pupae. Nonlinear regression was used to fit the variation trend of the days required for adult emergence with the changes in the time of pupae removed from winter conditions to the incubator.

## 3. Results

### 3.1. Ambient Temperature

**Figure 1:**
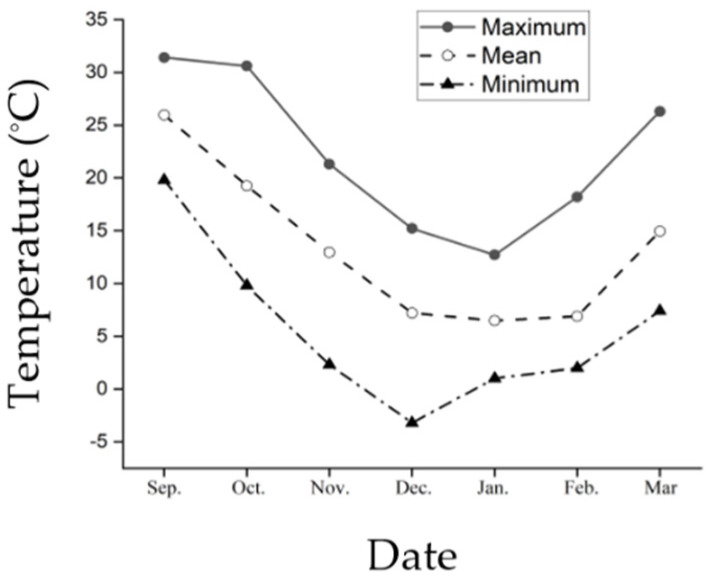
Seasonal change in maximum, mean and minimum ambient temperatures in the Huai’an area between September 2020 and March 2021.

The average ambient temperature in Huai’an area in China showed a decreasing and increasing trend with seasonal change, where the average ambient temperature was 25.9 °C in September, declining to 19.3 °C in October, 12.9 °C in November, 7.2 °C in December, 6.4 °C in January, and then increasing to 6.8 °C in February and 14.9 °C in March (Figure 1). The minimum temperature also showed a trend of decreasing and then increasing with seasonal change, with the minimum temperature dropping to −3.2 °C in December.

### 3.2. Supercooling Point

**Figure 2:**
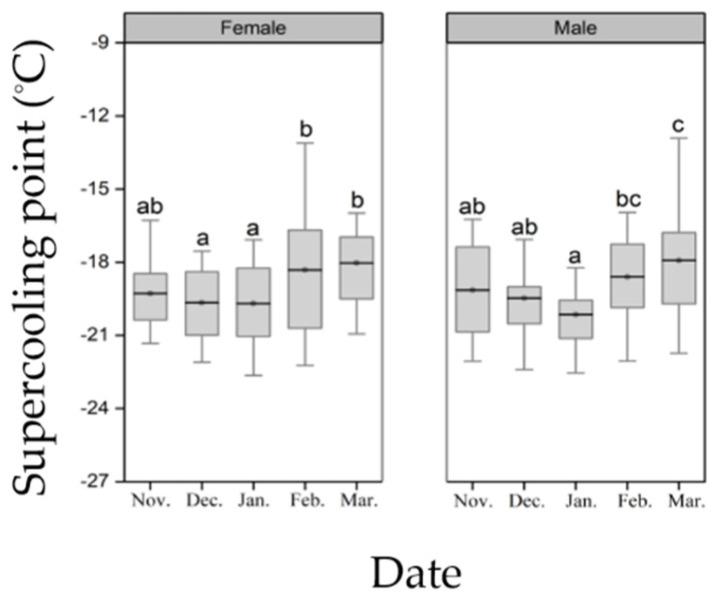
Seasonal change of supercooling point of *H. cunea* diapausing pupae. Different letters indicate significant differences from one another at *p* ˂ 0.05 (female: *n* = 16–19; male: *n* = 19–22). The top and bottom of each box represents the upper and lower quartile, respectively; the horizontal line represents the mean; the vertical lines extend to the minimum and maximum values within 1.5 times the inter-quartile range.

Seasonal changes had a significant effect on the supercooling point of diapausing pupae (female: F_4, 82_ = 3.02, *p* = 0.02; male: F_4, 99_ = 3.87, *p* = 0.0058, Figure 2). The supercooling point of female pupae were similar among the November, December and January treatment groups (*p* > 0.05), but the supercooling points in the February and March treatment groups were significantly higher than those of the December and January treatment groups (*p* < 0.05, Figure 2). Similar to female pupae, the supercooling points of male pupae in the November, December and January treatment groups were similar (*p* > 0.05), while the supercooling points of February and March were significantly higher than that of January (*p* < 0.05), and the supercooling points of March were significantly higher than those of the three treatment groups of November, December and January (*p* < 0.05, Figure 2).

### 3.3. Biochemical Analysis

**Figure 3:**
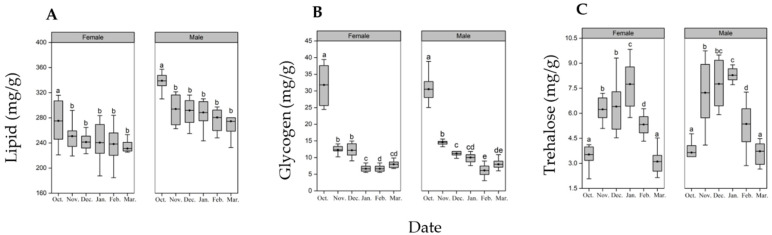
Seasonal patterns of biochemical composition of *H. cunea* diapausing pupae. (**A**): lipid, (**B**): glycogen, (**C**): trehalose. Different letters indicate significant differences from one another at *p* ˂ 0.05 (female: *n* = 11–12; male: *n* = 10–11). The top and bottom of each box represents the upper and lower quartile, respectively; the horizontal line represents the mean; the vertical lines extend to the minimum and maximum values within 1.5 times the inter-quartile range.

Seasonality had a significant effect on the lipid content of diapausing pupae (female: F_5, 66_ = 4.7, *p* < 0.001; male: F_5, 60_ = 9.4, *p* < 0.001, Figure 3A). The lipid content of female pupae showed a trend of first decreasing from October to November and then maintaining a stable level with seasonal change. The lipid content of female pupae decreased by 9.1% from October to November (*p* = 0.0012) and remained consistent from November to March (Figure 3A). We observed a similar trend in males, with lipid content decreasing 8.7 % (*p* = 0.0025) between October and November and then stabilizing from November to March (Figure 3A).

Seasonal change also had a significant effect on the glycogen content of diapausing pupae (female: F_5, 60_ = 151.83, *p* < 0.001; male: F_5, 60_ = 174.86, *p* < 0.001, Figure 3B). In female pupae, glycogen content decreased by 62 % between October and November (*p* < 0.001) and then deceased again between December and January (*p* < 0.001) before maintaining levels until March (Figure 3B). We observed a similar trend in male pupae, with a sharp decrease of 52% from October to November (*p* < 0.001). In males, glycogen content then continued to decrease through February to 33% of the initial glycogen content in October (Figure 3B).

Seasonal changes also had a significant effect on trehalose content of diapausing pupae (F_5, 60_ = 42.19, *p* < 0.001; F_5, 60_ = 31.75, *p* < 0.001, Figure 3C). Unlike lipid and glycogen content, which showed an obvious decrease with seasonal changes, diapausing pupae showed elevated levels of trehalose during colder months. From October to January, diapausing female pupae showed a sharp increase in trehalose content, with 1.76 times more trehalose in November than in October and reaching the maximum concentrations in January with 2.20 times trehalose relative to that in October. The trehalose content in February and March decreased sharply compared to January and was only 0.68 and 0.40 times of the trehalose content in January, respectively. Male pupae also showed a trend of increased trehalose content during colder months (Figure 3C). The trehalose content of male pupae in November, December and January was 1.98, 2.12 and 2.26 times of that in October, respectively, while that in February and March was 0.64 and 0.45 times of that in January, respectively.

### 3.4. Pupal Diapause Development Time and Adult Fitness of Post-Diapause

Premature removal of pupae from overwintering conditions had a significant effect on the development time of pupae (female pupae: F_3, 123_ = 288.99, *p* < 0.001; male pupae: F_3, 138_ = 263.94, *p* < 0.001, Table 1). Although there was no significant difference in the development time of pupae removed from winter conditions on 5 February and 25 February (*p* > 0.05), they were significantly shorter than the development time of pupae removed from overwintering conditions on 17 March, with the longest on 6 April (*p* < 0.05). The time of moving from outdoors to the incubator for heating also had a significant effect on the survival rate of pupae, decreasing significantly with the delay of moving to incubator. The pupae survival rates were similar in the 5 February and 25 February treatment groups (*p* > 0.05), but they were significantly higher than those in the 17 March and 6 April treatment groups, while that on the 17 March was significantly higher than that on 6 April (*p* < 0.05).

**Figure 4:**
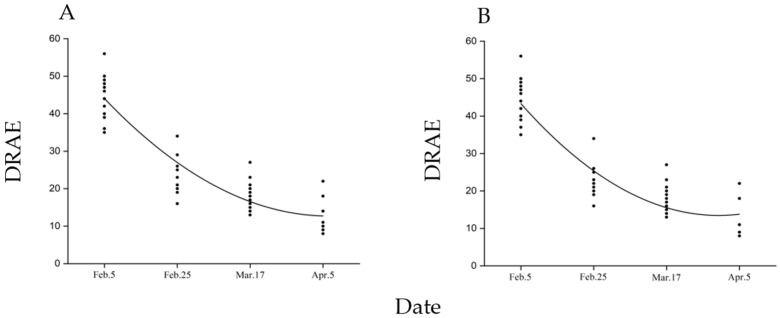
Effect of the time of pupae removed from winter conditions to the incubator on the time required for adult emergence. (**A**): female adult; (**B**): male adult. DRAE: The days required for adult emergence (female: *n* = 28–39; male: *n* = 25–34).

After diapausing pupae were moved from outdoors to the incubator and warmed, the time required for adult emergence decreased in a polynomial pattern (female: y = 0.0067x^2^ − 589.7x, R² = 0.8523, *p* < 0.01; male: y = 0.0088x^2^ − 783.41x, R² = 0.8501, *p* < 0.01, Figure 4). The time required for female and male emergence in the 5 February treatment group was 44.6 ± 5.3 d and 43.8 ± 5.3 d, respectively, shortening to 23.7 ± 4.6 d and 21.7 ± 3.9 d, respectively, by 25 February, 19.5 ± 4.2 d and 18.5 ± 4.2 d, respectively, by 17 March and only 12.9 ± 3.8 d and 13.1 ± 4.8 d by 5 April.

**Figure 5:**
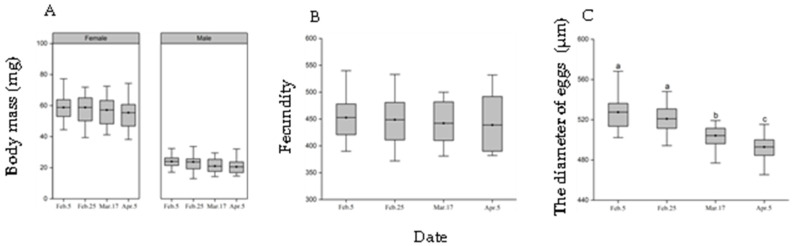
Effect of heating time inside the transfer chamber on the fitness of adults. (**A**): adult mass, (**B**): fecundity, (**C**): egg diameter. Different letters indicate significant differences from one another at *p* ˂ 0.05 (female body mass: *n* = 28–39; male body mass: *n* = 25–34; fecundity: *n* = 10; egg diameter: 27–35). The top and bottom of each box represents the upper and lower quartile, respectively; the horizontal line represents the mean; the vertical lines extend to the minimum and maximum values within 1.5 times the inter-quartile range.

The time of moving from outdoors to the incubator did not significantly affect adult body mass and female fecundity but had a significant effect on egg diameter (female body mass: F_3, 102_ = 0.55, *p* = 0.65; male body mass: F_3, 104_ = 2.72, *p* = 0.058; fecundity, F_3, 36_ = 0.16, *p* = 0.92; egg diameter: F = 39.05, *p* < 0.001, Figure 5). The diameter of eggs tended to decrease with warming time delay, with the diameter of eggs being 0.95, 0.93 times on 17 March, and being 0.96, 0.95 times on 5 April smaller than that of eggs on 5 February and 25 February, respectively.

## 4. Discussion

The results of Li et al., showed that *H. cunea* diapausing pupae under natural environmental conditions started to terminate diapause in March and this peaked in April [26]. The results of the present study are consistent with the results of Li et al. [26]. The time required for adult emergence was shortened with the delay of moving diapausing pupae back to incubation temperatures permissive to development, where the time required for adult emergence in the 17 March treatment group was slightly longer than that of non-diapausing pupae (14.0 days). Furthermore, the time required for adult emergence in the 5 April treatment group was similar to that of non-diapausing pupae. The results of Wang et al., (unpublished) showed that the metabolic rate of diapausing pupae on 2 March did not change significantly compared to that of diapausing pupae in December and January, so it was concluded that *H. cunea* diapausing pupae began to terminate diapause at the end of March under natural environmental conditions in the Huai’an area of China and this peaked in April.

Lipid is the most abundant energy reserve of diapause insects, which can meet the long-term energy demand of insects during diapause, and most non-feeding insects consume mainly lipids during the diapause period [34,35]. Our results are consistent with these previous findings; lipid content of the diapausing pupae decreased significantly after the beginning of diapause but did not change significantly in the late diapause period. These results suggest that the energy consumption of *H. cunea* diapausing pupae in the early stage was dependent on lipid. However, unlike our results, the lipid content of Asian tiger mosquito pupae, *Aedes albopictus*, did not change significantly during diapause [36], and the lipid content of the pistachio seed wasp *Eurytoma plotnikovi* Nikol’skaya even increased during diapause [37]. Therefore, there are interspecific differences in whether the energy consumption of insects in the early diapause period is lipid dependent.

Carbohydrates can also be used as an energy source during diapause. For example, energy consumption of *E. plotnikovi* diapause is dominated by carbohydrates [37]. Glycogen can be rapidly converted to glucose to meet limited energy requirements and also provide a source for the synthesis of cryoprotectants [6,11]. Our results showed that the glycogen content of diapausing pupae decreased sharply in the early diapause and then continued to decrease slowly throughout winter. The results of Zhao et al., also found that autumn warming accelerated carbohydrate consumption in diapausing pupae [38]. These results suggested that the early energy consumption of *H. cunea* diapausing pupae was also dependent on carbohydrates, while the energy metabolism of diapausing pupae was a dynamic process that changed from dependence on lipid and carbohydrates to carbohydrates or other energy substances as diapause progressed. Other insects such as *S. crassipalpis* show comparable results, with lipid consumption dominating early diapause and energy consumption shifting from dependence on lipid to carbohydrates or other energy substances in mid- to late diapause [10].

For overwintering insects, the supercooling point is an important indicator of cold hardiness; seasonal changes in cold hardiness is an important criterion for modeling changes in species distributions with changing climates [39]. The results of the current study showed that the diapausing pupae supercooling point did not change significantly when the ambient temperature decreased from November to January. Comparable results were obtained by Li et al., for diapausing *H. cunea* pupae in the Tsuruoka area of Japan [26]. However, the results of Xu et al., showed that the supercooling point of *H. cunea* diapause pupae from Northwest China showed a distinct seasonal variation pattern [27]. Together, these results suggest that the cold hardiness characteristic of *H. cunea* diapause pupae are variable within its invasive range in Asia.

Unlike the supercooling point, trehalose content increased with decreasing ambient temperature between October and January and started to decrease once the mean ambient temperature increased after February. Although the effect of low temperature on the survival rate of diapausing pupae was not examined in this study, the results of Li et al., showed that the survival rate of diapausing pupae under natural environmental conditions tended to decrease gradually from November to April, while low temperature acclimation helped to increase the trehalose reserves of pupae and helped to improve the cold hardiness [26]. The results of Izadi et al., on the Indian meal moth *Plodia interpunctella* (Hübner) and the locust bean moth *Ectomyelois ceratoniae* (Zeller) also showed that increased trehalose content helped to improve the cold hardiness of the insects [40]. Trehalose and glycerol are the main low temperature protectants for overwintering insects and trehalose appears to be the main contributor in *H. cunea* diapausing pupae, as glycerol was almost undetectable [26]. In the case of heat shock or freezing-induced dehydration, trehalose can effectively protect and stabilize cell membranes and protein structures [41], so this suggests that *H. cunea* diapausing pupae population in Jiangsu province improve their cold hardiness by increasing trehalose stores rather than decreasing their supercooling point.

The warming of the climate during diapause or the prolongation of diapause will increase the energy consumption of insects prior to spring emergence, which in turn will have a negative impact on the survival rate of diapausing insects or the fitness of individuals after diapause [42,43]. In areas with mixed bivoltine and trivoltine populations of *H. cunea*, the effect of a prolonged duration of diapause in second-generation populations increases the energy consumption during the diapause period, resulting in lighter pupae, reduced survival of overwintering pupae and reduced fecundity of adults during the diapause maintenance period compared to third-generation populations [44]. The results of Williams et al. [31] and Chen et al. [45] also showed that the survival rate of *H. cunea* pupae during diapause correlates with the duration of diapause; consistent with our results. A longer diapause period would increase the energy consumption of the insect, so it is speculated that the above results may be due to the increased energy consumption during the diapause period.

Williams et al. [31] showed that increased energy consumption during diapause negatively impacted post-diapause adult fecundity in *H. cunea*. Our results showed that an increase in pupal diapause development time, while increasing pupal mortality, had no significant effect on post-diapause adult body size and fecundity, but did negatively affect the egg diameter of post-diapause adults. Thus, we hypothesize that the increase in pupal diapause development time reduced the energy reserves of post-diapause adults and that adults adopted an adaptive strategy to cope with the reduced energy reserves by decreasing investment in egg production. Because egg diameter size is related to its resilience, the reduction in egg diameter of post-diapause adults may have a negative impact on the fitness of post-diapause adults. This remains to be verified by further experiments.

## 5. Conclusions

The energy consumption of *H. cunea* diapausing pupae was dominated by lipid and carbohydrates in the early stage of diapause and as diapause developed, the energy consumption of diapausing pupae shifted to glycogen or other energy stores. The trehalose content of *H. cunea* diapausing pupae showed an increasing and then decreasing trend with the change in ambient temperature, while the supercooling point did not decrease with the decrease in ambient temperature. The increase in pupae diapause development time had a significant negative effect on the survival rate of pupae during diapause and the fitness of adults after diapause.

## Figures and Tables

**Table 1:** Effect of time variation of moving from natural environmental conditions to incubator heating on the time from pupation to adult emergence and survival rate of pupae.

Time of Pupae Removed from Winter Conditions	Time from Pupation to Adult Emergence (d) (Mean ± SE)		Survival Rate %	
	Male Pupae	Female Pupae	Male Pupae	Female Pupae
5 February.	160.5 ± 4.4a	161.8 ± 4.4a	44.3a	46.2a
25 February	160.7 ± 3.9a	162.7 ± 4.6a	44.4a	46.1a
17 March	177.5 ± 4.1b	178.5 ± 4.1b	41.9b	44.6b
6 April	190.1 ± 4.2c	191.8 ± 3.8c	39.2c	42.0c

Different letters in the same column indicate significant differences between treatment groups (*p* < 0.05).

## Data Availability

The data presented in this study are available on request from the corresponding author.
